# Microbial genomic taxonomy

**DOI:** 10.1186/1471-2164-14-913

**Published:** 2013-12-23

**Authors:** Cristiane C Thompson, Luciane Chimetto, Robert A Edwards, Jean Swings, Erko Stackebrandt, Fabiano L Thompson

**Affiliations:** 1 Institute of Biology, Federal University of Rio de Janeiro (UFRJ), Rio de Janeiro, Brazil; 2 San Diego State University (SDSU), 5500 Campanile Drive, GMCS 411, San Diego, CA 92182, USA; 3 Ghent University, K. L. Ledeganckstraat 35, 9000 Gent, Belgium; 4 Leibniz Institute DSMZ-German Collection of Microorganisms and Cell Cultures, Braunschweig, Germany

**Keywords:** Microbes, Taxonomy, Genomics, Evolution

## Abstract

A need for a genomic species definition is emerging from several independent studies worldwide. In this commentary paper, we discuss recent studies on the genomic taxonomy of diverse microbial groups and a unified species definition based on genomics. Accordingly, strains from the same microbial species share >95% Average Amino Acid Identity (AAI) and Average Nucleotide Identity (ANI), >95% identity based on multiple alignment genes, <10 in Karlin genomic signature, and > 70% *in silico* Genome-to-Genome Hybridization similarity (GGDH). Species of the same genus will form monophyletic groups on the basis of 16S rRNA gene sequences, Multilocus Sequence Analysis (MLSA) and supertree analysis. In addition to the established requirements for species descriptions, we propose that new taxa descriptions should also include at least a draft genome sequence of the type strain in order to obtain a clear outlook on the genomic landscape of the novel microbe. The application of the new genomic species definition put forward here will allow researchers to use genome sequences to define simultaneously coherent phenotypic and genomic groups.

## Introduction

Microbial taxonomy comprises the identification of isolates into known species, the classification of new isolates (creation of new taxa), and nomenclature. While rules of the nomenclature are laid down in the Code of Nomenclature of Bacteria [1] the taxonomic schemes used for identification and classification need to be reliable, reproducible and informative. It is also desirable that the schemes are easy and affordable for the end-users of taxonomy. The phenotype-based taxonomy developed in the first half of the last century lead to a large multiplication of new species because very few features (colony morphology, physiologic aspects) were used as diagnostic for new taxa description. A complete revision in the number of recognized species was inevitable. More than 90% of all the species described in the Bergey’s Manual (1957) were subsequently reduced and only species included on the Approved Lists of Bacterial Names [2] became validly named species. Specifically, the major change in the schemes for species assessment occurred due to the development of DNA-DNA hybridization (DDH) and the introduction of the polyphasic taxonomy [3]. The methodology simply relies on the physico-chemical properties of DNA of homolog and hybrids in order to determine genetic distance (reassociation values and ΔTm). Molecular fingerprinting (e.g. rep-PCR, AFLP, and PFGE), and DNA sequencing completed the set of molecular tools necessary to establish and develop the polyphasic taxonomy in solid better robust underpins [4]. Taxonomic schemes still today based on the polyphasic approach that includes measures of evolutionary relationships using the gene sequences (most notably 16S rRNA gene), in order to determine the phylogenetic position of an isolate, combined with chemotaxonomic, physiological and cultural properties to assess novelty [5].

### The traditional microbial species delineation

A bacterial species is defined as a group of strains (including the type strain), having > 70% DDH similarity, < 5°C ΔTm, < 5% mol G + C difference of total genomic DNA, > 98% 16S rRNA identity [6]. The bacterial species definition is pragmatic and operational, aiming at the establishment of a rapid, reliable, reproducible, and useful taxonomic framework based on microbial evolution [7]. This polyphasic definition is a consensus in microbiology, although it is not based on a concept (i.e. the biological processes behind speciation and species). It is crucial to highlight that the current polyphasic framework does not question if this definition corresponds to a biological reality [8]. Within the framework of polyphasic taxonomy, strains of the same species have similar phenotypes (*e.g*. expression of different types of enzymes, ability to using different types of compounds as energy source, and growth in different temperatures and concentrations of acid and salt), genotypes (*e.g*. rep-PCR and AFLP), and chemotaxonomic features (*e.g*. FAME and polyamines), forming distinguishable tight groups [8]. Ideally, these groups should be readily identifiable and differentiated from closely related species.

The advent of whole genome sequencing (WGS) allowed the establishment of taxonomic schemes based on the evolutionary information contained in the genome sequences, such as the Karlin genomic signatures, Average Amino Acid Identity (AAI), supertrees, and *in silico* Genome-to-Genome Distance Hybridization (GGDH) [5]. Current sequencing technologies have become affordable to be used in routine microbial identification [9,10]. It is becoming clear that bacterial species can be defined on the basis of genomic signatures [11-15]. It is plausible to consider that microbial taxonomy will be steadily more dependent on genome sequences than relying on the classic polyphasic, including phenotypic characterization using time-consuming laborious laboratory tests. The current microbial taxonomy will now rapidly switch to genomic microbial taxonomy.

### The underpines of microbial genomic taxonomy

Whole-genome sequencing (WGS) launched microbial taxonomy into the new era of genomic microbial taxonomy [16], with the possibility of establishing systematics on the basis of information retrieved from complete genomes. The genomic microbial taxonomy will not merely be an enriched polyphasic taxonomy as it will be framed on a fundamental genomic backbone. The genomic taxonomy is defined on the basis of an integrated comparative genomics approach that include, e.g., the analysis of multilocus sequence analysis (MLSA), supertree analysis, average amino acid identity (AAI), average nucleotide identity (ANI), genomic signatures, codon usage bias, metabolic pathway content, core and pan genome analysis. The main goal of the genomic taxonomy is to extract taxonomic information from WGS that can be used to establish a solid framework for the identification and classification of prokaryote species and even populations. While evolutionary biology studies may be mainly concerned with the validity of the Domains of Life, frequency and quality of horizontal transfer and genomic plasticity events that may obfuscate the phylogenetic structure of the Domains of Life [17], population genomics studies may be mainly interested in microevolution and events that may lead to speciation or the appearance of highly successful populations [18]. Obviously, the first are interested in events on extremely large time scale events, whereas the second are concerned with contemporary events. Genomic taxonomy tries to embrace and connect these two study fields and is mainly concerned with species discovery and delineation.

Pioneering computational and mathematical studies performed in the 1990s [19-21], and confirmed by contemporaneous studies [22-28] suggested that genomes contain species-specific signatures. Genome signature is a compositional parameter reflecting the di-, tri-, or tetranucleotide relative abundance, which is similar between closely related species, and dissimilar between non-related species. Dinucleotide relative abundances (ρ*_XY_) is calculated using the equation ρ*_XY_ = f_XY_/f_X_f_Y_ where f_XY_ denotes the frequency of dinucleotide _XY_, and f_X_ and f_Y_ denote the frequencies of _X_ and _Y_, respectively [20,21]. The difference in genome signature between two sequences is expressed by the genomic dissimilarity (δ*), which is the average absolute dinucleotide of relative abundance difference between two sequences. The dissimilarities in relative abundance of dinucleotides between both sequences are calculated using the equation described by Karlin et al. (1997) [20]: δ*(f,g) = 1/16Σ| ρ*_XY_ (f) - ρ*_XY_ (g)| (multiplied by 1000 for convenience), where the sum extends over all dinucleotides. From this perspective, the set of dinucleotide biases constitutes a genomic signature that can discriminate sequences from different organisms. The dinucleotide biases appear to reflect species-specific properties of DNA stacking energies, modification, replication, and repair mechanisms [21]. WGS also permit the reconstruction of more robust taxonomic trees (*i.e*. supertrees) based on whole-genome sequence alignment of all genes of the core genome [29-31]. A good congruence was obtained by the traditional 16S rRNA based trees and the novel supertree method [29-31]. In the same period of time, computational biology studies performed on the diversity of virus in the early 2000s provided some clues for the potential use of whole genome sequences in the taxonomy of virus [32].

A virus species is a group of viruses that constitute a replicating lineage and occupy a particular ecological niche. In general, size and shape of the capsule and tail are critical features to define virus species, as well as genome size and type (e.g., single-stranded RNA [ssRNA], ssDNA, double-stranded RNA [dsRNA], and dsDNA) [33]. However, a new phage taxonomic structure based on Average Amino acid Identity (AAI) of complete phage genomes was proposed by Rohwer & Edwards (2002) [32]. The new method grouped phages into taxa that predicted several aspects of phage biology and highlighted genetic markers useful for monitoring phage biodiversity. AAI is calculated based on conserved protein-coding genes between a pair of genomes which are determined by whole-genome pairwise sequence comparisons using the BLAST algorithm [34]. For these comparisons, all protein-coding genes from one genome were searched against all protein-coding genes of the other genome. The genetic relatedness between a pair of genomes is measured by the average amino acid identity of all conserved genes between the two genomes. This study also indicated that genome signatures, such as the AAI, could be widely used in microbial taxonomy, beyond phage taxonomy.

In a similar fashion as proposed for phages [32], Konstantinidis & Tiedje (2005), Konstantinidis et al. (2006) [35-37] showed that the average amino acid identity (AAI) and average nucleotide identity (ANI) could be used to distinguish species of prokaryotes. Subsequently, a close relationship between DDH and ANI was shown [38], which was reassuring for the more traditional microbial taxonomists. Richter & Rosselló-Móra (2009) [39] suggested that the ANI between a given pair of genomes is the best alternative for a gold standard to species identification, since it mirrors DDH closely. The resulting averages reflected the degree of evolutionary distance between the compared genomes, and a value of higher than 94% ANI could represent the DDH boundary of higher than 70%. In addition, the authors showed that the tetranucleotide signature analysis correlated with ANI and that can be of help in deciding when a given pair of organisms should be classified in the same species. Because DDH is still the gold standard in prokaryotic taxonomy, most of the previous studies have tried to demonstrate the correlation between new methodologies and the traditional DDH experiments [40]. However, the recent developments of standard operating procedures for calculating genome-to-genome distances based on high-scoring segment pairs and new computational tools that allow the digital DNA-DNA hybridization for microbial species delineation by means of genome-to-genome sequence comparison are significant advances in genomic taxonomy [41-43]. The genome distance is calculated using Genome-To-Genome Distance Calculator (GGDC) [43]. Distances between a pair of genomes are determined by whole-genome pairwise sequence comparisons using one out of six supported local-alignment programs available in the GGDC. For these comparisons, algorithms are used to determine high-scoring segment pairs (HSPs) for inferring intergenomic distances for species delimitation. The corresponding distance threshold can be used for species delimitation. Any distance value above the threshold can be regarded as indication that the two genomes analyzed represent two distinct species. Distances are calculated by (i) comparing two genomes using the chosen program to obtain HSPs/MUMs and (ii) inferring distances from the set of HSPs/MUMs using three distinct formulas. Next, the distances are transformed to values analogous to DNA–DNA Hybridizations (DDH). These DDH estimates are based on an empirical reference dataset comprising real DDH values and genome sequences. The regression-based DDH estimate uses parameters from a robust-line fit, whereas the threshold-based DDH estimate applies the distance threshold leading to the lowest error ratio in predicting whether DDH is >70% or <70%. It is now possible to determine the DDH similarity between two microbial strains by means of whole genome sequences. The growing evidence obtained by various studies demonstrates the usefulness of genomic taxonomy. Light has been shed on the taxonomic structure of various microbial groups as discussed in the coming section.

### Example 1: taxonomy and ecologic population structure of vibrios

Vibrios are ubiquitous Gammaproteobacteria in the aquatic environment and can be found in association with animal or plant hosts. Currently, there are 152 known vibrio species (113 *Vibrio* spp.; 24 *Photobacterium* spp.; 6 *Aliivibrio* spp.; 4 *Enterovibrio* spp.; 4 *Salinivibrio* spp.; 1 *Grimontia* sp.) (http://www.bacterio.cict.fr/index.html). Some species are animal (e.g. *V. coralliilyticus* and *V. shiloi*) or human (e.g. *V. cholerae, V. parahaemolyticus,* and *V. vulnificus*) pathogens, and others form mutualistic relationships (e.g. *V. fischeri*) [44]. Identification of vibrios has been based on MLSA [45,46], DDH [44], ΔTm [47], and more recently in whole genome sequences [11,12,48]. The identification of vibrios remains a difficult task particularly for some sister species, e.g. the pairs *V. cholerae* and *V. mimicus*, *V. coralliilyticus* and *V. brasiliensis*, and the *V. splendidus* group, because they have similar genomes and phenotypes. In spite of the genome similarity, these species can be recognized as different evolutionary units in nature [49]. We analyzed a set of 43 genomes and observed that all strains formed groups that resemble the formal species. We also observed that the vibrios were distributed in three major groups or genera (*Vibrio*, *Photobacterium* and *Aliivibrio*) [11]. Based on the new genomic taxonomy, a Vibrio species is defined as a group of strains that share > 95% DNA identity in MLSA and supertree analysis, > 96% AAI, < 10 Karlin genomic signature, and > 70% GGDH. Strains of the same species and species of the same genus form monophyletic groups on the basis of MLSA and supertree analysis. Haley et al. (2010) [50] used a genomic taxonomy approach for the description of the new species *V. metecus* and *V. parilis*. These new species are phylogenetically closely related to *V. cholerae* and *V. mimicus*, respectively.

In addition to species identification and classification, genomics may also aid in the population structure studies of vibrios [51,52]. For instance, the distinct ecologic populations of the *V. splendidus* group appear to be organized in ecotypes, some occupying the particles or zooplankton, others occupying the free-living fraction of the plankton [53]. Subtle genomic differences in specific loci, related to e.g. chitin utilization, may explain the preferences of certain populations for the plankton than for the water column. However, a closer taxonomic examination indicated that some populations recognized in those studies are actually different species within the *V. splendidus* group and in other species groups previously recognized by polyphasic approaches, suggesting the need to reconciliate nomenclatures in population genomics and taxonomy [53,54].

Genomics has enhanced our understanding on the population structure of well known bacterial pathogens. For instance, *V. cholerae* O1 strains originated from Ghana (Africa) and from the Brazilian Amazonia were closely related on the basis of the MLSA and genome sequences, suggesting that i. nearly identical populations of *V. cholerae* could inhabit simultaneously these two continents and ii. the possible spread of successful populations to different geographic regions [46,55]. Further to this observation, the study of 25 *V. cholerae* O1 and Non-O1 isolates related to massive epidemics in 2009 and 2010 in Nigeria suggested the occurrence of multidrug resistant atypical El Tor strains, with reduced susceptibility to ciprofloxacin and chloramphenicol, characterized by the presence of the SXT element, and specific loci (*gyrA*, *parC*, *rstR*, *ctxB*, and *tcpA*), indicating a vast pathogenic potential in this geographic area [56]. It is possible that the recent Nigeria outbreaks of 2009 and 2010 were determined by multidrug resistant atypical O1 El Tor and non-O1/non-O139 [56]. The typical El Tor strain, from the beginning of seventh cholera pandemic, is no longer epidemic/endemic in this country, similarly to what was observed in other countries in East Africa and Asia [57].

### Example 2: the genus *Mycoplasma* is paraphyletic

Mycoplasmas are one of the smallest and simplest prokaryotes (Tenericutes), having only the minimal cellular machinery required for self-replication and survival. They appear to have evolved from Gram-positive bacteria by a process of degenerative evolution towards genome reduction and the loss of a cell wall [58,59]. Currently, there are 124 *Mycoplasma* species. They are widespread in nature as parasites of humans, mammals, reptiles, fish, arthropods, and plants. They may be symbionts of isopods [60], songbirds [61], wild and reared fish [62], and deep sea Lophelia corals of Gulf of Mexico and Norwegian Fjords [63]. Many *Mycoplasma* species are pathogenic for humans, animals, plants, and insects [64]. In addition, mycoplasmas have been a problem as intracellular contaminants in human cell therapy, and in the animal (poutry and swine farming) production as pathogens. Thus, rapid diagnostics and identification of mycoplasmas is crucial for various activities. Since the 1970s, serology has been established as the most important and widely used tool for defining and identifying *Mycoplasma* species (ICSB Subcommittee on the Taxonomy of Mycoplasmatales, 1972; ICSB Subcommittee on the Taxonomy of Mollicutes, 1979, 1995;) [65]. Roughly and as a first attempt, serological characterization is in agreement with DDH data and with 16S rRNA sequence data. However, differentiation of closely related species using 16S rRNA gene sequence and even DDH is very difficult because some *Mycoplasma* species may have up to 100% 16S rRNA gene similarity and approx. 70% DDH similarity. For instance, the pairs *M. pneumoniae* and *M. genitalium*, *M. mycoides* and *M. capricolum* have 98% and 99.8% 16S rRNA sequence similarity, respectively. Serology is also very cumbersome, requiring special reagents and the expertise of very few international laboratories. The high genomic and phenotypic diversity of mycoplasmas may also result in cross-reaction or misidentification based on serology. In order to test the feasibility of the genomic taxonomy in mycoplasmas, several genomic markers were analyzed in a collection of 46 genomes. The availability of whole genome sequences of several closely related species, such as *M. pneumoniae* and *M. genitalium* formed an ideal test case for the establishment of the genomic taxonomy of Mycoplasmas. Disclosing species-specific patterns for the different genome-wide markers reinforced their usefulness in mycoplasma taxonomy.

*Mycoplasma pneumoniae* and *M. genitalium* had only 73% MLSA similarity, 67% AAI, and 88 for Karlin genomic signature. Codon usage of the phylogeneticaly distantly related species *M. conjunctivae* and *M. gallisepticum* was identical, in spite of clear differences in MLSA, AAI, and Karlin signature, suggesting that these two species were subjected to convergent adaptation due to similar environmental conditions. Similar to our observations in the study of the genomic taxonomy of vibrios, *Mycoplasma* species may be defined based on genomic features, such as DNA identity in MLSA, AAI and Karlin genomic signature. According to the phylogenetic reconstructions based on genome signatures, the genus *Mesoplasma* appeared to be nested within *Mycoplasma*, putting in question its taxonomic validity. The genera *Acholeplasma* and candidate *Phytoplasma* appeared at the outskirts of the phylogenetic tree, whereas the genus *Ureaplasma* formed a separated branch distinct from the genus *Mycoplasma*. The genus *Mycoplasma* appeared to be paraphyletic, indicating the power of the genomic analysis in order to refine and enhance the taxonomic structure of complex microbial groups.

### Example 3: a new taxonomic framework for the *Prochlorococcus* ecotypes

*Prochlorococcus* is globally abundant and dominates the total phytoplankton biomass and production in the global oligotrophic ocean. *Prochlorococcus* is currently one of the best studied marine microbes [66-69]. They can reach 3 × 10^5^ cells per mL in a wide range of environments from the bottom of the euphotic zones to the upper layer of the oligotrophic zones of the global ocean [68]. According to the temperature and other environmental parameters preferences, the *Prochlorococcus* are identified in at least six ecotypes. An ecotype is defined as a genetically coherent population within a species, having a subtle niche preference, different from other conspecific populations, Until recently, the taxonomic structure of the genus *Prochlorococcus* was poorly defined though. There was only one species formally described in this genus, named *P. marinus*, and two subspecies, *P. marinus* subsp *marinus* and *P. marinus* subsp *pastoris* (http://www.bacterio.cict.fr/index.html). In spite of the apparent mix of strains, the apparent high genomic similarity among the different types of known and unknown ecotypes, and the apparent unstable taxonomic structure, a closer examination of the currently available genomic data of *Prochlorococcus* allowed us to make significant progress in the taxonomy of this group.

**Figure 1:**
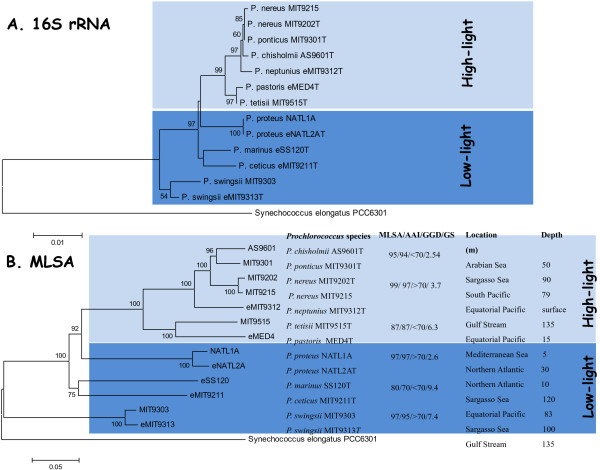
**Phylogenetic trees of the 16S rRNA gene (A) and MLSA (B) based on maximum likelihood method.** Distance estimation was obtained by the model of Kimura 2-Parameter. Bootstrap percentages after 2,000 replications are shown. Scale bar, estimated sequence divergence. *Synechococcus elongates* PCC6301 was used as an outgroup. MLSA tree: new *Prochlorococcus* species (^T^ type strains), MLSA (%), AAI (%), GGD (%) and Genome Signature (GS) values for each *Prochlorococcus* pair, location and depth are indicated on the right side of the tree. This figure is reproduced with permission from Thompson et al. [15].

We analyzed the 13 representative complete genome sequences of cultured *Prochlorococcus* strains and over 100 marine metagenomes in order to determine the taxonomic structure of the genus by means of several genomic taxonomy tools. In our hands, the current species *P. marinus* actually comprised 10 cryptic species (Figure 1). This newly established taxonomic framework for the genus *Prochlorococcus* was then used to identify metagenomic sequences. We observed that there may only be a total of 35 *Prochlorococcus* species in the world’s oceans, but some species (e.g. *Prochlorococcus chisholmii* AS9601^T^) are very abundant, whereas others (e.g. *Prochlorococcus swingsii* MIT9313^T^) appear to be restricted to fewer locations. The incongruence between the ecotype designation previously used in the *Prochlorococcus* and our newly proposed taxonomic framework is because the ecotype designation is given to populations within species with subtle niche preferences, whereas the species designation refers to organisms that have diversified a long time ago and thus have different genomes and phenotypes based on the criteria used for species circumscription [10,12]. In support of our view, several studies have advanced the utility of a genomic taxonomy for prokaryotic species delineation [54,70,71].

### Genotype to phenotype in microbial taxonomy

Phenotypic identification is critically important in different fields of microbiology because it provides the clues e.g. for treatment of infectious agent for humans, animals and plants, and for technological applications. It is becoming evident that phenotypic tables commonly found in new species descriptions and diagnostic manuals will be constructed using genome information to complement, or even replace the use of commercially available phenotypic tests (such as API and BIOLOG substrate panels) currently used in taxonomy. Though apt for many species, the commercial systems are rarely devised to characterize the broad spectrum of environmental strains. Because phenotypic features can be obtained directly from the genome sequences by means of the analyses of the presence or absence of diagnostic genes, it is now possible to derive diagnostic phenotypes from the genotypes in different taxonomic groups [15,72,73]. For instance, the species *V. cholerae* is positive for D-mannose and sucrose fermentation, Voges-Proskauer, and lipase activity, whereas its sister species, *V. mimicus* is negative according to genome sequence analysis [72]. Similarly, the phenotypic identification of *Prochlorococcus* was also performed based on the presence of diagnostic genes. As automated genome annotation tools (e.g. the RAST, Kbase, and Model SEED) progress, we will be able to automatically and accurately determine the major phenotypic characteristics of microbes from their genome sequences. Of course, complex metabolism may still require culture-based experiments.

### Concluding remarks

Microbial taxonomy is critically important for different fields, including medicine, agriculture, marine ecology and conservation [74]. A unified prokaryotic species definition based on genomics would consider that strains from the same species share <10 in Karlin signature, >95% AAI and ANI, >95% identity based on multiple alignment genes, and > 70% *in silico* GGDH. Species of the same genus will form monophyletic groups on the basis of 16S rRNA gene sequences, MLSA and supertree analysis. Some exceptions may occur, e.g. in the case of *Prochlorococcus* where different species may have nearly identical Karlin signatures, suggesting convergent evolution in this genomic feature. In spite of the possible exceptions, this definition should be widely applicable for different types of microbes. The recent developments in genomic prokaryotic taxonomy demonstrate their importance in the development of more ample and refined taxonomic schemes.

Taxa descriptions have increased sharply in the last ten years due to technological developments and the environmental surveys in different locations of the globe. The number of new species descriptions in the 1990s was 2,082, whereas in the last decade, 69 new families, 688 new genera, and 3,344 new species, were described [5]. Most of the descriptions are based on one strain and included the concomitant proposal of the higher ranks. Clearly, there is no sign of reduction in the species descriptions in the coming decade. However, the standards for species descriptions need to be refined and more unified. Some requirements and suggestions were discussed by an ad hoc committee, including ample phenotypic characterization, examination of several (at least five) strains, high quality 16S rRNA sequences (>1,300 nt, <1% ambiguous nt), DDH with closely related neighboring strains, and MLSA data [75]. In addition to all these requirements, we propose that new taxa descriptions should also include at least a draft genome sequence, with at least a 20X coverage, of the type strain, in order to obtain the majority of the genomic landscape of the novel bacterium. The genome sequence of the new taxa can be deposited in large public databases, such as the EMBL and GenBank, in order to allow assess by the scientific community and automatic identification of microbial species through the internet [11-15]. The application of the new genomic species definition and taxonomic frameworks put forward here will allow researchers to use genome sequences to define phenotypically and genomically coherent and cohesive groups.

## Competing interests

The authors declare that they have no competing interests.

## Authors’ contributions

All authors contribute equally to this commentary. All authors read and approved the final manuscript.
